# Lysyl oxidase family members in urological tumorigenesis and fibrosis

**DOI:** 10.18632/oncotarget.24948

**Published:** 2018-04-13

**Authors:** Tao Li, Changjing Wu, Liang Gao, Feng Qin, Qiang Wei, Jiuhong Yuan

**Affiliations:** ^1^ The Andrology Laboratory, West China Hospital, Sichuan University, Chengdu, Sichuan, China; ^2^ Department of Urology, West China Hospital, Sichuan University, Chengdu, Sichuan, China; ^3^ Department of Urology, The Second Affiliated Hospital of Chongqing Medical University, Chongqing, China

**Keywords:** lysyl oxidase, tumorigenesis, fibrosis, urological cancer, collagen

## Abstract

Lysyl oxidase (LOX) is an extracellular copper-dependent monoamine oxidase that catalyzes crosslinking of soluble collagen and elastin into insoluble, mature fibers. Lysyl oxidase-like proteins (LOXL), LOX isozymes with partial structural homology, exhibit similar catalytic activities. This review summarizes recent findings describing the roles of LOX family members in urological cancers and fibrosis. LOX/LOXL play key roles in extracellular matrix stability and integrity, which is essential for normal female pelvic floor function. LOX/LOXL inhibition may reverse kidney fibrosis and ischemic priapism. LOX and LOXL2 reportedly promote kidney carcinoma tumorigenesis, while LOX, LOXL1 and LOXL4 suppress bladder cancer growth. Multiple studies agree that the LOX propeptide may suppress tumor growth, but the role of LOX in prostate cancer remains controversial. Further studies are needed to clarify the exact effects and mechanism of LOX/LOXL on urological malignancies.

## LYSYL OXIDASE

Lysyl oxidase (LOX) is a copper-dependent monoamine oxidase in the extracellular matrix (ECM) [1]. There are four LOX isozymes in humans that share partial structural homology with LOX, namely lysyl oxidase-like proteins 1–4 (LOXL1/2/3/4), and the five members together form the lysyl oxidase family [1, 2]. The four LOXLs share 85%, 58%, 65%, and 62% homology with LOX, respectively [2], and the human LOX/LOXL genes are located on chromosomes 5, 15, 8, 2, and 10, [1, 2]. LOX/LOXLs are expressed at different levels, and human LOX expression is low under normal conditions. While overall LOXL2/3/4 expression is low in adult rats, LOXL1 levels are higher than those of LOX [3]. The human LOX/LOXL proteins also vary greatly in size; LOX has the shortest open reading frame, encoding 417 amino acids, while LOXL2 has the longest, with 774 amino acids [2, 3] (Table 1).

**Table 1:** Lysyl oxidase family member

Member	Human chromosome	mRNA size	Protein size	Similarity to LOX	Highest mRNA levels in adult tissue distribution
LOX	5	4.8 kb	417 AA	-	Heart, lung, kidney, skeletal muscle
LOXL1	15	2.4 kb	574 AA	85%	Heart, lung, pancreas, spleen, skeletal muscle
LOXL2	8	4.0 kb	774 AA	58%	Lung, thymus, testis, ovary, skin
LOXL3	2	3.3 kb	753 AA	65%	Heart, uterus, testis, ovary
LOXL4	10	3.5 kb	756 AA	62%	Pancreas, testis, skeletal muscle

LOX: lysyl oxidase; LOXL: lysyl oxidase like protein; AA: amino acids.

Both LOX and LOXL have catalytic activities. The C-terminus regions of the five family members share 95% homology, and are comprised of a highly conserved, 205-amino acid structural domain that is essential for bioactivity [3]. This catalytic region includes four conserved histidine residues to coordinate copper binding and a cofactor lysine tyrosine quinone (LTQ) formed by conserved lysine and tyrosine residues [3]. The NH_2_-terminal sequence is unique to each family member, and may determine cellular localization and/or mediate protein-protein interactions [3].

LOX/LOXL expression is highly tissue-specific. LOX is most commonly expressed in the heart and is also highly expressed in lung, kidney, testis, uterus, and placenta. LOXL1 is abundant in heart, lung, kidney, pancreas, and muscle. LOXL2 is abundant in the early stages of fetal heart development, and is highly expressed in the uterus, placenta, and other organs [2–4]. However, these are lowly expressed in brain and liver [4]. LOX3 is also detected in the heart, uterus, testis, and ovary [2, 3] (Table 1).

LOX is initially synthesized as a 46 kDa pro-zymogen by fibroblasts and myofibroblasts. Signal peptide shearing and N-terminal glycosylation convert the pro-zymogen into the 50 kDa LOX proenzyme, which is inactive or has limited activity [2, 3]. The proenzyme then undergoes post-translational modifications in the endoplasmic reticulum and Golgi apparatus, and is transferred into extracellular spaces. Here, extracellular proteolytic enzymes shear the proenzyme at a Gly-Asp bond into two peptide segments of sizes 30 and 18 kDa, the mature LOX form and LOX-pp (LOX pro-peptide), respectively [5, 6].

## LOX BIOLOGICAL FUNCTION

### Collagen crosslinking

LOX can catalyze the oxidative deamination of lysine and hydroxylysine residues of certain non-helical telopeptide regions in collagen molecules to generate lysine- or hydroxylysine-aldehyde. Condensation reactions then spontaneously occur between the aldehydes and neighboring lysine or hydroxylysyl residues, producing divalent, crosslinked dehydro-hydroxylysinonorleucine (HLNL) and dehydro-dihydroxylysinonorleucine (DHLNL) [2, 7, 8]. DHLNL reacts with the third lysine or hydroxylysyl residue to generate mature crosslinked products, pyridinoline and/or deoxy-pyridinoline [2, 7, 8]. As this process continues, HLNL and DHLNL concentrations decrease, while that of pyridinolines and other mature products increase during early growth and development of the aorta [7]. At present, pyridinoline is used to detect mature collagen fibers [2, 7].

### Elastin crosslinking

LOX oxidatively deaminates lysine residues in elastin to lysine-aldehydes. After a series of spontaneous condensation reactions, three reactive lysine-aldehydes and the fourth lysine residue form tetrafunctional desmosines, which are also used to measure mature elastin levels [7, 9].

The most important biological activity of LOX is catalyzing crosslinking of soluble collagen or elastin proteins in the ECM into insoluble mature fibers. This process is essential for ECM structural stability and integrity, and contributes to tissues tensile strength [10, 11].

### Tumorigenesis

LOX/LOXL may promote or suppress tumorigenesis, depending on cell type, location, and transformation status [12, 13]. LOX expression is enhanced in various tumors, including head and neck squamous cell carcinoma (HNSCC), and breast and colorectal cancers [11, 12], and is associated with poor disease-free and overall survival [10]. Factors that promote tumor development or progression, such as TNF-α, TGF-β, or HIF-1, can upregulate LOX [10, 13]. LOX also reportedly improved tumor cell proliferation and survival through focal adhesion kinase (FAK) activation, which subsequently upregulates fibronectin and provides a permissive niche to support metastasis [13].

LOXL2 remodels the tumor microenviroment, regulates cell adhesion, invasion, and motility, and improves tumor cell survival and chemoresistance [12]. LOXL2 overexpression was correlated with more aggressive breast cancer [14, 15], and primary gastric tumor invasion, lymph node metastasis, and reduced patient survival. LOXL2 may promote tumorigenesis and progression by activating Src/FAK signaling, which can be suppressed using a LOXL2 antibody [16].

LOXL4 is upregulated in HNSCC, likely via the transcription factors, TATA-box-binding protein (TBP) and SP1, which bind the LOXL4 promoter [17]. LOXL4 also promotes colorectal tumor metastasis and resistance to radiotherapy and chemotherapy [12, 18].

These studies suggest that LOX/LOXL promote cancer progression. However, LOX was initially described as a tumor suppressor, by inhibiting HRAS-mediated oncogenic transformation [12]. LOX downregulation has been observed in tumor tissues, and silencing LOX was associated with a more aggressive tumor phenotype and decreased patient survival [10]. This tumor suppressive activity is associated with 18 kDa LOX-pp, which inhibited FGF-2 signaling in prostate cancer (PCa) [19], oncogenic bcl-2 activity in breast cancer [20], and nuclear factor-κB (NF-κB) activation in lung and prostate carcinoma [10]. LOX-pp also promoted phenotypic reversion in ras-transformed NIH3T3 cells [21], and promoted apoptosis in PCa cells by blocking mitogen-activated protein kinase/extracellular signal-related kinase pathways [22] or inhibiting DNA repair [23].

## LOX REGULATION

**Figure 1:**
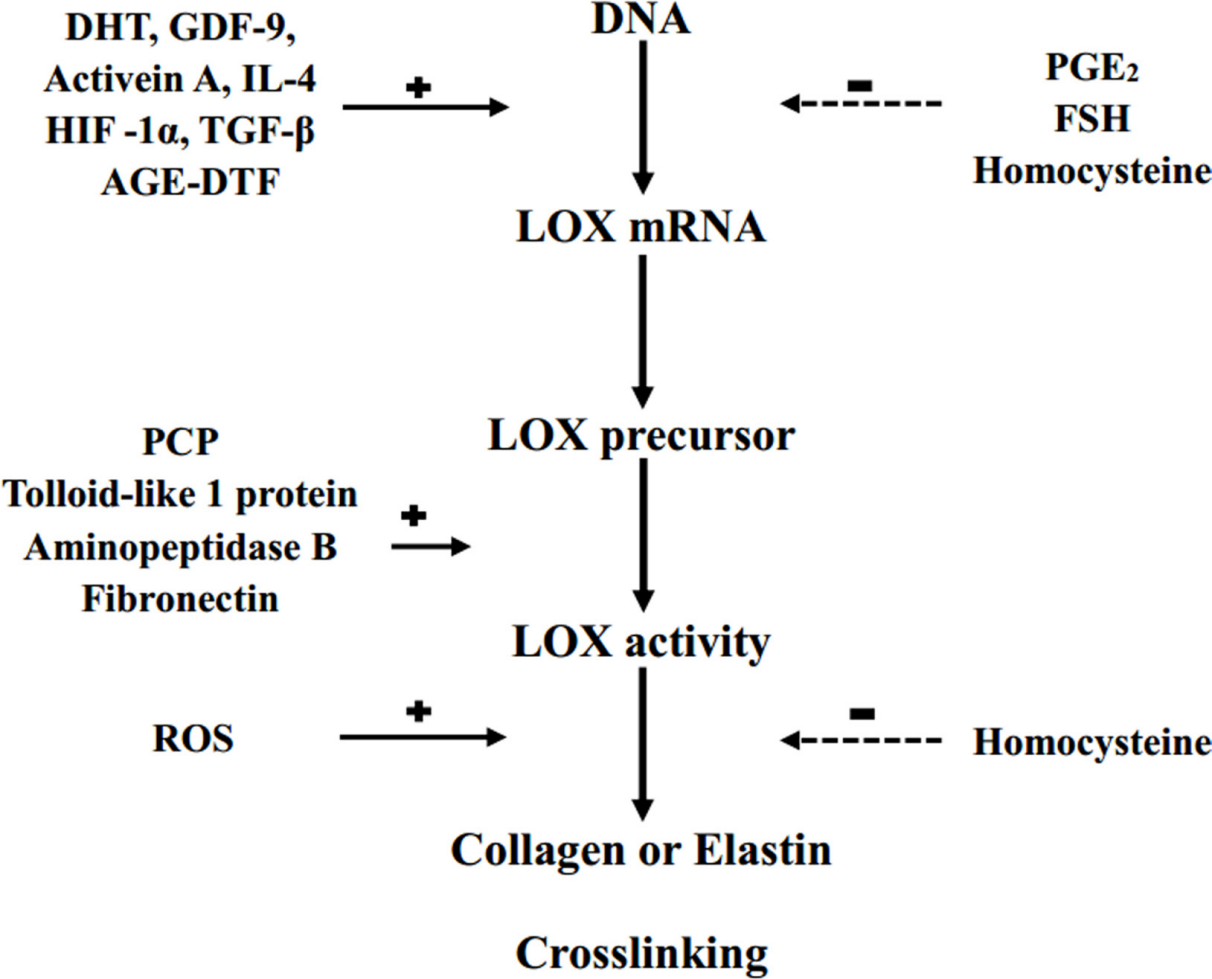
LOX regulation DHT: dihydrotestosterone; GDF-9: growth differentiation factor-9; IL-4: interleukin-4; HIF-1α: hypoxia inducible factor 1α; TGF-β: transforming growth factor-β; AGE-DTF: advanced glycation end products-dependent transcription factor; PCP: procollagen enzyme C; ROS: reactive oxygen specie; PGE2: prostaglandin E2; FSH: follicle-stimulating hormone; +: stimulation; –: inhibition.

LOX is regulated at three levels: LOX precursor synthesis in myofibroblasts and fibroblasts, extracellular conversion to the mature enzyme, and direct regulation of enzymatic activity [2]. The many LOX regulators include promoters of dihydrotestosterone (DTH), growth differentiation factor-9 (GDF-9), Activen A [24], interleukin-4 (IL-4) [25], hypoxia inducible factor 1α (HIF-1α), advanced glycation end products-dependent transcription factor (AGE-DTF), transforming growth factor-β (TGF-β), procollagen enzyme C (PCP), tolloid-like protein 1, fibronectin, aminopeptidase B, and reactive oxygen species (ROS) [2, 26–28], as well as inhibitors of follicle-stimulating hormone (FSH) [24], prostaglandin E_2_ (PGE_2_), and homocysteine [2, 29] (Figure 1).

The irreversible LOX inhibitor, β-aminopropionitrile (BAPN), is currently used in research both *in vivo* and *in vitro* [7, 30]. However, lathyrism is a long-term side effect of BAPN, and includes bone deformities, joint dislocation, kyphoscoliosis, weakening of tendon and ligament attachments, weakening of skin and cartilage, hernia formation, and arterial aneurysm or dissection [30].

## LOX IN THE URINARY SYSTEM

### Kidney

LOX/LOXL2 overexpression in human clear cell renal cell carcinoma (ccRCC) [31, 32] promoted tumor cell migration and adhesion, as well as matrix stiffness to enhance tumor progression and metastasis [32]. LOXL2 overexpression was associated with higher ccRCC pathological stages, and upregulated integrins-α5/β1, which enhanced cancer cell survival, invasion, and metastasis via protease- and proteasome-dependent mechanisms [31]. Tumor suppressive microRNA-26a/b [33] and microRNA-29s [34] directly inhibited LOXL2 to reduce RCC cell migration and invasion. These findings suggest new strategies for treating ccRCC [32] (Table 2).

**Table 2:** Summarise of LOX/LOXL on uro-oncological researches

Kidney carcinoma			
Am J Pathol, 2016, [32]	Di Stefano V	1. LOX promoted ccRCC progression and metastasis by increasing cellular migration, adhesion, stiffness of matrix increment.	LOX Promoter
Mol Cancer Res, 2014, [31]	Hase H	1. LOXL2 was correlated with ccRCC pathological stage. 2. LOXL2 promoted ccRCC progression by improving integrins-α5/β1.	LOXL2 Promoter
Int J Oncol, 2016, [33]	Kurozumi A	1. MicroRNA-26a/b inhibited LOXL2 to restrain ccRCC migration and invasion.	LOXL2 Promoter
FEBS Lett, 2015, [34]	Nishikawa R	1. MicroRNA-29 inhibited LOXL2 to restrain ccRCC migration and invasion.	LOXL2 Promoter
**Bladder carcinoma**			
Oncotarget, 2016, [41]	Gross-Cohen M	1. Tumor suppressor of heparanase 2 is related to low cancer grading and staging. 2. Bladder carcinomas which exhibited strong Hpa2 staining also showed strong LOX staining	LOX Suppressor
Cancer Res, 2007, [40]	Wu G	1. LOXL1/4 gene methylation and loss of expression was found in primary bladder cancer. 2. LOXL1/4 inhibited Ras/ERK pathway to exert suppress bladder cancer.	LOXL1/4 Suppressor
Mol Cancer, 2014, [42]	Deng H	1. MicroRNA-193a-3p promote bladder cancer chemoresistance via repressing LOXL4 expression.	LOXL4 Suppressor
**Prostate cancer (PCa)**			
Oncol Lett, 2016, [46]	Zheng W	1. LOX status was higher in low-grade PCa than high-grade tissue. 2. Decreased LOX level increased risk of tumor associated mortality.	LOX Suppressor
Cancer Res, 1998, [47]	Ren C	1. LOX mRNA was decreased in metastatic tumor than primary prostate tumor. 2. LOX expression decreased during progression of metastasis	LOX Suppressor
PLoS One, 2015, [48]	Nilsson M	1. High LOX expression in tumor adjacent non-malignant prostate epithelium were associated with shorter cancer specific survival. 2. Strong LOX level in prostate tumor epithelium was correlated to higher Gleason score and metastases.	LOX Promoter
Sci Rep, 2016, [49]	Nilsson M	1. Administration of BAPN, inhibitor of LOX, before AT-1 cells implantation suppressed PCa growth.	LOX Promoter
J Hum Genet, 2017, [50]	Kato M	1. LOXL2 was overexpressed in PCa region. 2. LOXL2 knockdown inhibited PCa migration and invasion.	LOXL2 Promoter
Oncogene, 2009, [19]	Palamakumbura AH	1. LOX-pp inhibited DNA synthesis, ERK1/2, AKT and FRS-2α to suppress proliferation of PCa.	LOX-pp Suppressor
Oncogene, 2015, [23]	Bais MV	1. LOX-pp induce nuclear DNA repair foci to make PCa sensitive to radiation effect.	LOX-pp Suppressor
J Cell Commun Signal, 2016, [52]	Alsulaiman M	1. rLOX-pp inhibit OPG but enhance CCN2 expression to stimulate osteoclast fusion	LOX-pp Suppressor

Mild atrophy and varying degrees of fibrosis have been observed in the tubulointerstitial tissues of spontaneously hypertensive rats, with TGF-α/β upregulation and LOX downregulation in the renal cortex compared to control rats with normal blood pressure. High dose perindopril could reduce hypertensive renal injury by antagonizing mineralocorticoid to prevent LOX downregulation [35]. However, both TGF-β1 and LOX were overexpressed in the tubular epithelial cells of a hereditary nephritis mouse model with chronic renal fibrosis. Elevated LOX can catalyze interstitial collagen crosslinking to induce irreversible chronic renal tubulointerstitial fibrosis in kidney [36]. Adriamycin toxicity may also be the result of LOX upregulation leading to this irreversible progression [37]. Similarly, hyperuricemia-induced LOX upregulation can increase fibronectin synthesis in tubular epithelial cells and promote renal fibrosis, and LOX knockdown via siRNA reduces this effect [38]. Additionally, transplanted renal parenchyma exhibiting delayed graft failure after 6–12 months showed increased LOX activity, which could induce ischemia, renovascular hypertension, renal dysfunction, and failure [39].

### Bladder

LOXL1/4 gene methylation and loss of expression is commonly observed in primary bladder cancer cells, and LOXL1/4 reintroduction into these cells can reduce colony formation. LOXL1/4 might suppress cancer cell growth by blocking Ras/ERK signaling [40]. The tumor suppressor, heparanase 2 (Hpa2), attenuates bladder carcinoma and is associated with lower tumor grading and staging. Cells with high Hpa2 expression also overexpress LOX. By enhancing LOX expression, Hpa2 might reduce Erk phosphorylation to suppress bladder tumor growth [41]. Additionally, microRNA (miR)-193a-3p modulates oxidative stress (OS) signaling to promote multi-drug chemoresistance in bladder cancer by inhibiting LOXL4 expression. The miR-193a-3p/LOXL4/OS axis may be a new target for anti-bladder cancer chemotherapeutics [42] (Table 2).

Menkes disease (MD), an infantile-onset X-linked recessive neurodegenerative disorder, is characterized by copper (Cu) metabolism deficiency or dysfunction [43]. LOX enzymatic activity, which is essential for collagen or elastin crosslinking, can be affected by copper transport alterations. LOX deficiency-related connective tissue defects are accompanied by a high frequency of bladder diverticula (38.6% of total patients) [43]. Moreover, tissue inflammation was noted in a bladder outlet obstruction (BOO) model, with elevated LOX expression in lamina propria and increased collagen secretion in isolated urothelial cells [44]. Hypoxia was also triggered in partial BOO rats. While 2-methoxyestradiol reduced HIF-1α expression in these rats, LOX, downstream of HIF-1α, was substantially elevated. This suggests the presence of additional hypoxia or inflammatory stimuli that can induce LOX overexpression [45].

### Prostate

LOX expression is lower in PCa tissues than in benign prostate hyperplasia (BPH) nodules [46], and is also reduced in metastases compared with primary PCa tissues, suggesting LOX decline during progression to metastasis [47]. Lower LOX level was associated with higher PCa grade and increased risk of tumor-associated mortality [46]. These findings suggest that LOX acts as a tumor suppressor in PCa.

On the contrary, another group found that high LOX expression in adjacent non-malignant prostate epithelium was associated with shorter cancer-specific survival, and LOX overexpression in tumor epithelium was related to higher Gleason score and metastasis [48]. Administration of the LOX inhibitor, BAPN, before AT-1 cell implantation suppressed PCa growth in an animal model, while treatment started after tumor implantation had no effect, or even increased cell growth [49]. Thus, LOX function appears context-dependent, exhibiting both tumor promoting and suppressing properties in PCa [49]. Further studies are needed to clarify the circumstances under which LOX inhibitors could be adapted as anti-PCa therapeutics.

Knockdown of highly expressed LOXL2 in PCa can reduce tumor cell invasion and migration [50].

Meanwhile, LOX-pp appears to act as a tumor suppressor in lung, breast, pancreas, and gastrointestinal system cancers [51–53]. In PCa, elevated autocrine fibroblast growth factor 2 (FGF-2) promotes tumor cell growth by stimulating DNA synthesis, ERK1/2, AKT and FRS-2α (fibroblast growth factor receptor substrate-2). However, LOX-pp blocks these pathways to reduce cell proliferation (DU 145), suggesting LOX-pp targeting of the FGF receptor [19]. Recombinant LOX-pp (rLOX-pp) induced nuclear DNA repair, increasing PCa cell sensitivity to ionizing radiation [23]. rLOX-pp also decreased osteoprotegerin (OPG) and increased CCN2 expression in bone marrow-derived cells to improve osteoblast differentiation, which was inhibited by PCa cell-conditioned media [52]. Thus, simultaneous administration of recombinant LOX-pp and LOX inhibitors may be a promising anti-cancer treatment option [10, 47]. Further research addressing the contrasting roles of mature LOX/LOXL and LOX-pp in cancer progression, and a better understanding of LOX-associated signaling in tumorigenesis will be required for the success of such therapeutics [10, 47] (Table 2).

### Testis

One study showed that LOXL1 plays a subtle and likely multifactorial role in male fertility and sexual development [54]. High LOXL3/4 was observed in human testis, although their exact roles are presently unknown [55, 56].

### Other

LOX expression was low, and collagen fiber arrangements were loose and disorganized in the pelvic floor ligaments of female patients with stress urinary incontinence [57]. LOX downregulation can decrease collagen crosslinking and increase the susceptibility of immature collagen to degradation, which may reduce pelvic floor ligament tensile strength or elasticity [57]. However, reduced ECM synthesis, increased proteolysis, and LOX overexpression were observed in urethral cells from a rat stress urinary incontinence model after vaginal trauma [58]. Trauma can elevate LOX expression, which may explain this discrepancy

Female pelvic floor dysfunction is closely associated with elastin stability [59]. Normal elastin crosslinking in the urogenital tract was hampered in LOXL1 knockout mice, which had higher frequencies of pelvic organ prolapse (POP) [59] and lower urinary tract dysfunction (LUTS), as well as lower mean bladder capacities and voiding pressures [60]. In these mice, POP incidence was more frequent in spontaneous than cesarean delivery models, with higher bladder contraction frequencies and more disorganized elastic fibers in the vagina and urethra [61]. However, further investigation is needed to characterize underlying mechanisms.

Overexpressed LOX may also promote nephrolithiasis [62]. Additionally, our previous work showed that LOX accelerated corpus cavernosum fibrosis in an ischemic priapism rat model, while BAPN reversed the fibrotic process [63].

## CONCLUSIONS

LOX family members induce varying effects, including tumor promotion and inhibition, in different urological organs. While normal LOX/LOXL expression is crucial for female pelvic floor function, LOX/LOXL inhibitors might reverse kidney fibrosis and ischemic priapism. Further investigation is necessary to clarify the specific functions of LOX/LOXL in tumorigenesis and progression.
